# Comparative In Vitro Study: Assessing Phytochemical, Antioxidant, Antimicrobial, and Anticancer Properties of *Vaccinium macrocarpon* Aiton and *Vaccinium oxycoccos* L. Fruit Extracts

**DOI:** 10.3390/pharmaceutics16060735

**Published:** 2024-05-29

**Authors:** Rima Šedbarė, Valdimaras Janulis, Alvydas Pavilonis, Vilma Petrikaite

**Affiliations:** 1 Department of Pharmacognosy, Faculty of Pharmacy, Lithuanian University of Health Sciences, 50162 Kaunas, Lithuania; valdimaras.janulis@lsmu.lt; 2 Department of Analytical and Toxicological Chemistry, Faculty of Pharmacy, Lithuanian University of Health Sciences, 50161 Kaunas, Lithuania; 3 Institute of Microbiology and Virology, Veterinary Academy, Lithuanian University of Health Sciences, 47181 Kaunas, Lithuania; alvydas.pavilonis@lsmu.lt; 4 Laboratory of Drug Targets Histopathology, Institute of Cardiology, Lithuanian University of Health Sciences, 50162 Kaunas, Lithuania; vilma.petrikaite@lsmu.lt

**Keywords:** cranberry, antioxidant activity, antimicrobial activity, anticancer activity, spheroids

## Abstract

The phytochemical diversity and potential health benefits of *V. oxycoccos* and *V. macrocarpon* fruits call for further scientific inquiry. Our study aimed to determine the phytochemical composition of extracts from these fruits and assess their antioxidant, antibacterial, and anticancer properties in vitro. It was found that the ethanolic extracts of *V. oxycoccos* and *V. macrocarpon* fruits, which contained more lipophilic compounds, had 2–14 times lower antioxidant activity compared to the dry aqueous extracts of cranberry fruit, which contained more hydrophilic compounds. All tested cranberry fruit extracts (OE, OW, ME, and MW) significantly inhibited the growth of bacterial strains *S. aureus*, *S. epidermidis*, *E. coli*, and *K. pneumoniae* in vitro compared to the control. Cytotoxic activity against the human prostate carcinoma PPC-1 cell line, human renal carcinoma cell line (CaKi-1), and human foreskin fibroblasts (HF) was determined using an MTT assay. Furthermore, the effect of the cranberry fruit extract samples on cell migration activity, cancer spheroid growth, and viability was examined. The ethanolic extract from *V. macrocarpon* fruits (ME) showed higher selectivity in inhibiting the viability of prostate and renal cancer cell lines compared to fibroblasts. It also effectively hindered the migration of these cancer cell lines. Additionally, the *V. macrocarpon* fruit extract (ME) demonstrated potent cytotoxicity against PPC-1 and CaKi-1 spheroids, significantly reducing the size of PPC-1 spheroids compared to the control. These findings suggest that cranberry fruit extracts, particularly the ethanolic extract from *V. macrocarpon* fruits, have promising potential as natural remedies for bacterial infections and cancer therapy.

## 1. Introduction

The cranberry is a plant of the *Ericaceae* family, belonging to the *Vaccinium* genus [1]. Two types of cranberry species are usually distinguished: large cranberry (*Vaccinium macrocarpon* Aiton) and small cranberry (*Vaccinium oxycoccos* L.) [1]. The two species of cranberry differ in morphological characters such as leaf length, leaf shape, pedicel bract length, and pedicel bract shape [2].

Many researchers have focused their research on *V. macrocarpon*, a species native to the eastern region of North America, whose fruits are grown commercially [2]. The literature provides research results on the effects of bioactive compounds in *V. macrocarpon* fruits [3]. The documented antimicrobial activity of bioactive compounds in *V. macrocarpon* fruits has popularized the use of cranberry juices and extracts for treating and preventing urinary tract infections [3,4,5]. Cranberry products are available in various forms (juices, supplements, and extracts) for urinary tract health [4,6].

The geographical range of small cranberries is much wider than that of large cranberries. *V. oxycoccos* grows naturally in various wetlands in Europe, Asia, and North America [7,8]. Morphologically, *V. oxycoccos* fruits are smaller compared to *V. macrocarpon* [9]. Small cranberry fruits are commonly used in juice drinks, jams, jellies, sauces, and additives in meat products [10]. Although the fruit of *V. oxycoccos* is widely used in folk medicine as well as in the food and pharmaceutical industry, research on its bioactive compounds and their effects on biological activity remains limited compared to the extensive studies conducted on large cranberry fruits [8].

Complexes of biologically active compounds have been identified in the fruits, which determine the various pharmacological effects of cranberry fruits [11,12]. Phenolic compounds, including flavonoids and phenolic acids, are the predominant compounds in cranberry fruit [12]. These compounds have antioxidant properties that help combat oxidative stress and thus reduce the risk of chronic diseases such as cardiovascular illness and cancer [13,14,15]. The proanthocyanidins found in large cranberry fruits have been studied for their anti-adhesive properties, which prevent bacteria from adhering to the lining of the urinary tract, reducing the risk of urinary tract infections [4]. In addition, large and small cranberry fruits contain triterpenoids, which have anticancer activity [16,17]. Cranberry fruit extracts inhibit the proliferation of cancer cells and induce apoptosis, which has made them an important object of research [11,18].

Studies of the qualitative and quantitative composition of secondary metabolites in the fruits of *V. macrocarpon* and *V. oxycoccos* and the prediction of their biological effects are a very important area of research in evaluating the quality and efficacy of their products. The phytochemical composition of small and large cranberry fruits is influenced by genetic and environmental factors, and the composition of their extracts is determined by the extraction conditions and applied extraction methods [19,20]. It was found that the profiles of anthocyanins, flavonols, and triterpene compounds in small cranberry and large cranberry fruit samples were quite similar, but the quantitative composition of bioactive compounds in cranberry fruits varies [21,22,23]. Jungfer et al. found that the concentration of A-trimeric proanthocyanidins in *V. macrocarpon* fruits was up to 15 times higher than in *V. oxycoccos* fruits [24]. Differences in the qualitative and quantitative composition of the compounds between the two *Vaccinium* species may influence their biological activity. Further research in this area is needed to better understand the health benefits of small and large cranberries [16,24].

The aim of our study was to estimate the phytochemical composition of *V. oxycoccos* and *V. macrocarpon* fruit extracts and to determine their antioxidant, antibacterial, and anticancer activities in vitro. These studies enable the comparison of the effects of extracts from different cranberry species on inhibiting the growth of bacterial strains (*Staphylococcus aureus*, *Staphylococcus epidermidis*, *Proteus vulgaris*, *Klebsiella pneumoniae*, *Pseudomonas aeruginosa*, and *Escherichia coli*) and their cytotoxicity against human prostate carcinoma (PPC-1) and renal carcinoma (CaKi-1) cell lines. Studying the biological effects of *V. macrocarpon* and *V. oxycoccos* fruit extracts is important for determining their potential health benefits and discovering potential new compounds.

## 2. Materials and Methods

### 2.1. Preparation of Cranberry Fruit Dry Extracts

Dry *V. oxycoccos* (OE) and *V. macrocarpon* (ME) fruit extracts were obtained by ethanol extraction. Small and large cranberry fruit samples were frozen at −20 °C and then transferred to a −60 °C low-temperature freezer (CVF330/86, ClimasLab SL, Barcelona, Spain). The frozen cranberry fruits were dried in a freeze-drier (Zirbus Technology GmbH, Bad Grund, Germany) at a pressure of 0.01 mbar and a condenser temperature of −85 °C. The freeze-dried cranberry fruits were crushed using an electric mill (Retsch GM 200, Retsch GmbH, Hahn, Germany). Freeze-dried large cranberry fruit powder and freeze-dried small cranberry fruit powder were extracted with 96% (*v*/*v*) ethanol (AB Stumbras, Kaunas, Lithuania) at a 1:50 ratio in a percolator. The collected ethanolic extract of cranberry fruit was distilled to remove the ethanol. The distilled extract was freeze-dried at a pressure of 0.01 mbar at a condenser temperature of −85 °C. The freeze-dried ethanolic extract of *V. oxycoccos* (OE) and *V. macrocarpon* (ME) fruit was then ground to powder.

Dry *V. oxycoccos* (OW) and *V. macrocarpon* (MW) fruit extracts obtained by hot water extraction. The fruits of large cranberries and small cranberries were cut and extracted with hot water by maceration at a 1:2 ratio. The aqueous cranberry fruit extract was lyophilized at a pressure of 0.01 mbar and a condenser temperature of −85 °C. The freeze-dried aqueous extract of *V. oxycoccos* (OW) and *V. macrocarpon* (MW) fruit was then ground to powder.

### 2.2. Chromatographic Analysis

The qualitative and quantitative composition of anthocyanins, phenolic compounds, and triterpenoids in cranberry fruit was analyzed utilizing a Waters ACQUITY Ultra High-Performance LC system (Waters, Milford, MA, USA) equipped with a photodiode array detector. Separation of compounds was achieved using an ACE C18 reversed-phase column (ACT, Aberdeen, UK; dimensions: 100 × 2.1 mm, particle size: 1.7 µm).

The qualitative and quantitative composition of anthocyanins and anthocyanidins in cranberry fruit samples was determined using a methodology developed and validated by Vilkickytė et al. [25]. The column temperature was maintained at 30 °C. Gradient elution was conducted using mobile phase A (aqueous 10% formic acid (Merck, Darmstadt, Germany) solution) and mobile phase B (acetonitrile (Sigma-Aldrich, Steinheim, Germany)) according to the following protocol: 0.0–2.0 min, 95% A; 2.0–7.0 min, 91% A; 7.0–9.0 min, 88% A; 9.0–10.0 min, 75% A; 10.0–10.5 min, 20% A; 10.5–11.0 min, 20% A; and 11.0–12.0 min, 95% A. The flow rate was maintained at 0.5 mL/min with an injection volume of 1 µL. Detection was performed at a wavelength of 520 nm using a PDA detector.

The analysis of the composition of flavonols in cranberry fruit was performed using the methodology developed and validated by Urbštaitė et al. [26]. A gradient consisting of 100% acetonitrile (solvent A) and an aqueous solution of 0.1% (*v*/*v*) formic acid (solvent B) was used to partition the flavonols. The gradient variation was as follows: 0 min, 5% B; 1 min, 12% B; 3 min, 12% B; 4 min, 13% B; 9 min, 25% B; 10.5 min, 30% B; 12 min, 30% B; 12.5 min, 90% B; 13 min, 90% B; 13.5 min, 5% B; and 14.5 min, 5% B, with the delay of the next injection being 2 min. The injection volume was 1 μL, the flow rate was 0.5 mL/min, and the column temperature was 30 °C. The flavonols detected were quantified at the 360 nm wavelength.

The determination of triterpene compounds in cranberry fruit was performed using the methodology developed and validated by Šedbarė et al. [22]. The gradient consisted of an aqueous solution of 0.1% (*v*/*v*) formic acid (solvent A) and 100% methanol (Sigma-Aldrich, Steinheim, Germany) (solvent B). The gradient variation was as follows: 0 min, 92% B; 8 min, 97% B; 9 min, 98% B; 29.5 min, 98% B; and 30 min, 92% B, with the delay of the next injection being 10 min. The injection volume was 1 μL, the flow rate was 0.2 mL/min, and the column temperature was 25 °C. The triterpene compounds and phytosterols detected were quantified at the 205 nm wavelength.

### 2.3. Spectrophotometric Studies

The total amount of proanthocyanidins was determined using the DMCA (4-dimethylaminocinamaldehyde) method [27]. Ten microliters of cranberry extract were mixed with 3 mL of a 0.1% DMCA (Sigma-Aldrich, Steinheim, Germany) reagent solution in acidified ethanol (9:1, *v*/*v*). The absorption was measured after 5 min at λ = 640 nm using an M550 UV/Vis spectrophotometer (Spectronic CamSpec, Garforth, UK), with the DMCA solution in acidified ethanol serving as the reference solution. The total proanthocyanidin content was calculated from the calibration curve of (−)-epicatechin (0.0625 mg/mL^−1^ mg/mL) (*y* = 0.7021*x* + 0.0138; R^2^ = 0.9994) and expressed as µg/g (−)-epicatechin equivalent (EE) dry weight.

### 2.4. Determination of the Antioxidant Activity In Vitro

The ABTS^∙+^ radical cation scavenging assay was carried out using the methodology described by Raudone et al. [28]. The in vitro determination of the antiradical activity by the TFPH radical cation scavenging assay was carried out using the methodology for the determination of antioxidant activity described by Asghar et al. [29]. The in vitro determination of the reducing activity via the FRAP technique was performed using the methodology for the determination of the reducing activity described by Raudone et al. [30]. The in vitro determination of the reducing activity via the CUPRAC method was performed using the methodology for determining the reducing activity described by Ozyurek et al. [31]. The antioxidant activity of cranberry fruit extracts was evaluated in three independent tests.

### 2.5. Determination of Antimicrobial Activity In Vitro

The antimicrobial activity was tested on the following strains of microorganisms: *Staphylococcus aureus* (ATCC 25923), *Staphylococcus epidermidis* (ATCC 12228), *Proteus vulgaris* (ATCC 8427), *Klebsiella pneumoniae* (ATCC 13883), *Pseudomonas aeruginosa* (ATCC 27853), and *Escherichia coli* (ATCC 25922). The strains were inoculated diagonally on Mueller-Hinton II Agar (BBL, Cockeysville, MA, USA) plates and cultured at 37 °C for 12 h. The microorganisms were suspended in a sterile saline solution (0.85%). The turbidity of the microorganism suspension in the test tube was 0.5 according to the McFarland standard (Ref. SD2300, Pro-Lab Diagnostics, Bromborough, UK). One milliliter of the prepared suspension was mixed with 10 mL of agar (at 40 °C) in a Petri dish (diameter 10 cm) in an even layer over the bottom of the dish. Stainless steel hollow cylinders of 8 mm in diameter were placed on top of the solidified agar. The inside of the cylinders was filled with 100 μL of the test cranberry extract dissolved in 0.85% saline (15 mg/mL). The control samples were prepared by filling the cylinders with 100 μL of 0.85% saline. Petri dishes were incubated in a KB8182 incubator (Termaks, Bergen, Norway) at 37 °C for 24 h. After the incubation, the diameters of the inhibition zones around the cylinders were measured (in mm). Three independent replications of the study were carried out.

### 2.6. Determination of the Anticancer Activity In Vitro

The human prostate carcinoma PPC-1 cell line was obtained from Prof. Tambet Teesalu (University of Tartu, Tartu, Estonia). The human renal cell carcinoma (CaKi-1) cell line was obtained from the American Type Culture Collection (Manassas, VA, USA). Human foreskin fibroblasts (HF) CRL-4001 were obtained from Prof. Helder Santos (University of Helsinki, Helsinki, Finland). The effect of cranberry extracts on cell viability was determined via the MTT assay [32].

We determined the effect of cranberry fruit extracts on cell migration activity [33]. Prostate carcinoma (PPC-1) and renal carcinoma (CaKi-1) cells were seeded in 24-well plates in 500 μL of cell culture medium and were incubated at 37 °C in a humidified atmosphere containing 5% CO_2_ until cells formed a monolayer. Then, the monolayer was scratched with a sterile 100-μL pipette tip at the center of each well. After that, the old medium was replaced with a fresh medium containing cranberry extracts, with the concentration being at previously established EC_50_ and EC_10_ values. Prostate carcinoma (PPC-1) “wounds” were photographed every 12 h, and renal carcinoma (CaKi-1) “wounds” were photographed every 24 h. The “wound” area was analyzed using the ImageJ software (version 1.54i, National Institutes of Health, Bethesda, MD, USA).

We determined the effect of cranberry fruit extracts on 3D cell cultures (spheroids) [34]. Spheroids were formed from PPC-1, CaKi-1 cells, and fibroblasts by the magnetic 3D bioprinting method. PPC-1, CaKi-1, and human fibroblasts at 70% confluency in a 6-well plate were incubated with Nanoshuttle (n3D Biosciences, Inc., Houston, TX, USA) for 8 h at 37 °C in a humidified atmosphere containing 5% CO_2_. The cells were detached using TrypLE reagent (Gibco), centrifuged, and then seeded into ultra-low attachment 96-well plates at a density of 1.5 × 10^3^ cancer cells and 1.5 × 10^3^ human fibroblasts per well in a volume of 100 μL. The plate was placed on a magnetic drive and incubated for 2 days at 37 °C in a humidified atmosphere containing 5% CO_2_. Then the fresh medium containing 1 mg/mL of the cranberry extract was introduced into the wells.

Photos of spheroids were taken every two days using the Olympus IX73 inverted microscope (OLYMPUS CORPORATION, Tokyo, Japan). The effect of the cranberry extracts on 3D PPC-1 and CaKi-1 cells was analyzed by measuring the diameter change of spheroids using ImageJ software (National Institutes of Health, Bethesda, MD, USA).

On the last day of incubation, 10 μL of WST-1 reagent (Sigma-Aldrich Co., St. Louis, MO, USA) was introduced to each well with spheroids. Following a 10-h incubation, 50 μL of liquid from each well was transferred to a new 96-well plate. The absorbance was assessed at 460 and 530 nm using a multi-detection microplate reader, and cranberry extract effects on spheroid cell viability were calculated.

The anticancer activity using the MTT assay, the magnetic 3D bioprinting method, and the “wound healing” method were determined in three independent replicates.

### 2.7. Statistical Analysis

Data analysis was carried out using SPSS Statistics 29 (SPSS Inc., Chicago, IL, USA) and Microsoft Excel 2021 (Microsoft, Redmond, WA, USA). During the study, the arithmetic means and standard deviations of three replicated tests were calculated. The Kruskal–Wallis one-way ANOVA (for k samples) test with multiple comparisons was used to assess whether the differences between the groups of comparisons were statistically significant (*p* < 0.05).

## 3. Results and Discussion

### 3.1. In Vitro Study of the Phytochemical Composition and Antioxidant Activity of Cranberry Fruit Extracts

Recently, many studies have been carried out to identify compounds with protective antioxidant properties in plant matrices and to test their application for preventive purposes [35,36]. The antioxidant properties of phenolic and triterpene compounds identified in plant matrices have been linked to biological effects such as anti-inflammatory, antitumor, and antimicrobial activity [37]. Thus, it is relevant to determine the qualitative and quantitative composition of the bioactive compounds in *V. macrocarpon* and *V. oxycoccos* fruit extracts and to evaluate the potency of their antioxidant activity.

In this study, we determined the qualitative and quantitative phytochemical composition of the dry extracts of *V. macrocarpon* and *V. oxycoccos* fruit samples (Table 1). Dry ethanolic extracts of *V. oxycoccos* (OE) and *V. macrocarpon* (ME) fruit samples were found to contain triterpenoids (7126 ± 106 µg/g and 4084 ± 61 µg/g, respectively) and anthocyanidin aglycones (1648 ± 20 µg/g and 3787 ± 42 µg/g, respectively), as well as, albeit in lower amounts, anthocyanin glycosides (582 ± 9 µg/g and 390 ± 7 µg/g, respectively), flavonols (48 ± 0.4 µg/g and 169 ± 2 µg/g, respectively), and chlorogenic acid (86 ± 0.5 µg/g and 7 ± 0.1 µg/g, respectively) (Table 1). No proanthocyanidins were detected in these dry extracts of cranberry fruit samples (Table 1). Aqueous extracts of *V. oxycoccos* (OW) and *V. macrocarpon* (MW) fruit samples were found to contain proanthocyanidins (1531 ± 46 µg EE/g and 1359 ± 119 µg EE/g, respectively), chlorogenic acid (1897 ± 19 µg/g and 458 ± 5 µg/g, respectively), flavonols (2287 ± 20 µg/g and 2865 ± 26 µg/g, respectively), and anthocyanins (4721 ± 66 µg/g and 6165 ± 86 µg/g, respectively) (Table 1). No lipophilic triterpene compounds were detected in the dry aqueous extracts of cranberry fruit samples (OW or MW) (Table 1).

The comparison of the ethanolic extracts of *V. oxycoccos* (OE) and *V. macrocarpon* (ME) showed that the OE extract had a higher content of triterpenoids and chlorogenic acid, while the ME extract had a higher content of anthocyanidin aglycones. The comparison of the aqueous extracts of *V. oxycoccos* (OW) and *V. macrocarpon* (MW) revealed that the OW extract had a 4-fold higher content of chlorogenic acid, a similar total content of proanthocyanidins, a similar total content of flavonols, and a lower content of anthocyanins compared to the MW extract.

**Figure 1:**
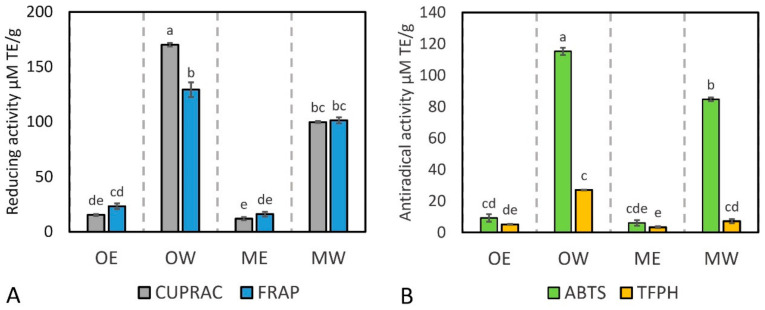
Determination of the reducing (**A**) and antiradical (**B**) activity of dried cranberry fruit extracts. OE—dry *V. oxycoccos* fruit extract obtained by extraction with 96% (*v*/*v*) ethanol; OW—dry *V. oxycoccos* fruit extract obtained by extraction with hot water; ME—dry *V. macrocarpon* fruit extract obtained by extraction with 96% (*v*/*v*) ethanol; MW—dry *V. macrocarpon* fruit extract obtained by extraction with hot water. Statistically significant differences between the antioxidant activity of cranberry extracts are indicated by different letters (*p* < 0.05).

In order to gain a deeper understanding of the antioxidant potential of the cranberry fruit extracts tested, their antiradical activity was evaluated using the ABTS and TFPH assays, and their reductive activity was evaluated using the FRAP and CUPRAC assays (Figure 1).

The aqueous extract of cranberry fruit samples (OW) was found to have the strongest reducing activity (170 µM TE/g) as tested via the CUPRAC method. Meanwhile, aqueous extracts of fruit samples of *V. macrocarpon* (MW) and *V. oxycoccos* (OW) tested via the FRAP technique were found to have the strongest reducing activity (101 µM TE/g and 129 µM TE/g, respectively), and there was no statistically significant difference in antioxidant activity between these extracts. The aqueous extract of *V. oxycoccos* fruit samples (OW) showed the strongest antiradical activity as assessed via the ABTS (115 µmol TE/g) and TFPH (27 µmol TE/g) methods (Figure 1).

In plant materials, it is difficult to attribute the biological effects to a specific group of compounds, as the complex of polyphenolic compounds acts synergistically [37,38]. Liu et al. found that triterpene compounds have lower antiradical activity, as assessed by the ABTS and DPPH methods, compared to phenolic compounds [39]. The antioxidant activity is thus closely related to the content of phenolic compounds [40]. The stronger antioxidant properties found in aqueous extracts OW and MW than in ethanolic extracts OE and ME were attributed to the higher content of phenolic compounds, which determined the stronger antioxidant activity under in vitro conditions.

Cranberries are rich in flavonoids and other phenolic compounds that may contribute to their antioxidant activity [41,42]. The antioxidant properties of these compounds are closely related to their structure [43]. The antioxidant activity is influenced by the number and position of hydroxyl groups, the presence of a double bond, glycosylation, and other functional groups [43]. Borges et al. found that phenolic compounds in cranberry fruit are responsible for the antioxidant activity, which was assessed via the HPLC-AOC method [44]. The results showed that the percentage antioxidant activity of the phenolic compounds identified in cranberry fruit samples was as follows: anthocyanins—39%, proanthocyanidin dimers—12%, flavonols—10%, and chlorogenic acid—2% [44]. The authors pointed out that the polymeric proanthocyanidins were either retained on the HPLC column or eluted, and thus their antioxidant activity was not assessed, but it is likely that their antioxidant activity was likely more significant than what was found during the study [44]. The proanthocyanidins detected in cranberry fruit samples are mainly composed of epicatechin units and therefore have more hydroxyl groups in their structure, which results in antioxidant properties [45,46]. Proanthocyanidins can act as hydrogen donors, accepting free radicals and generating free radicals themselves, forming stable intramolecular hydrogen bonds with free radicals, and thus blocking free radical chain reactions [47].

The significantly higher content of chlorogenic acid found in the aqueous extract of *V. oxycoccos* fruit (OW) than in the other extracts may have contributed to the enhancement of the antioxidant activity. Borges et al. reported that chlorogenic acid accounts for 2% of the antioxidant activity of the compounds identified in cranberry fruit extract [44]. The natural antioxidant properties of chlorogenic acid are due to its molecular structure, which contains five active hydroxyl groups and one carboxyl group [48].

Cranberry fruit extracts contain a high proportion of phenolic compounds such as anthocyanins, anthocyanidins, and flavonols, which have a significant effect on antioxidant activity. Oszmiański et al. found that the anthocyanin content and flavonol content of cranberry fruit samples correlated with the antioxidant activity of cranberry fruit extracts as determined via the ABTS, FRAP, and DPPH techniques [49]. Similar findings have been reported by other authors as well [41].

Cranberry ethanolic extracts (OE and ME) contained higher levels of anthocyanidins than glycosides. Vuolo et al. pointed out that the antioxidant activity of glycosides is weaker than that of the corresponding aglycones, as the latter can directly interact with free radicals [43]. The anthocyanidin aglycon content of *V. macrocarpon* ethanolic extract ME was found to be higher than the aglycon content of *V. oxycoccos* ethanolic extract OE, but this did not have a significant effect on the antioxidant activity, as the antioxidant activity of OE and ME extracts was not statistically significantly different.

The synergistic action of the biologically active compounds identified in the cranberry fruit samples determined the antioxidant activity of the ethanol extracts (OE and MW) and aqueous extracts (OW and MW) of the cranberry fruit were tested. The different extraction methods chosen significantly affected the phytochemical composition of the dry extracts and led to differences in the antioxidant activity of the dry extracts.

### 3.2. In Vitro Antibacterial Activity of Cranberry Fruit Extracts

Since the phytochemical composition of the bioactive compounds in *V. oxycoccos* and *V. macrocarpon* cranberry fruits varies, it is relevant to carry out comparative studies on the antimicrobial activity of the different cranberry extracts to gain a more detailed insight into the antimicrobial potential of the two cranberry species and to assess their potential health-promotion benefits.

**Figure 2:**
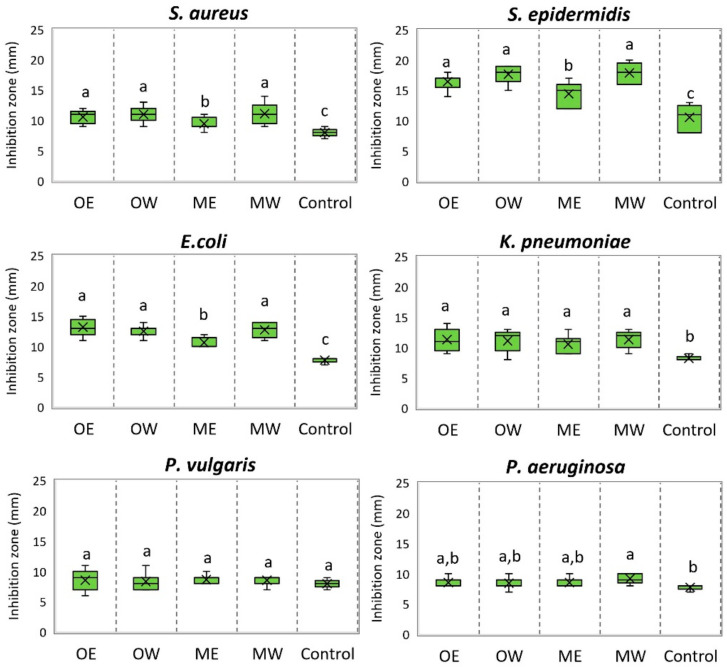
Determination of the antimicrobial activity of dried cranberry fruit extracts. OE—dry *V. oxycoccos* fruit extract obtained by extraction with 96% (*v*/*v*) ethanol; OW—dry *V. oxycoccos* fruit extract obtained by extraction with hot water; ME—dry *V. macrocarpon* fruit extract obtained by extraction with 96% (*v*/*v*) ethanol; MW—dry *V. macrocarpon* fruit extract obtained by extraction with hot water. Statistically significant differences between the antibacterial activity of cranberry extracts are indicated by different letters (*p* < 0.05).

The antibacterial activity of dry ethanolic (OE and ME) and aqueous (OW and MW) cranberry fruit extracts was determined by the treatment of strains of bacterial cultures of *S. aureus*, *S. epidermidis*, *E. coli*., *P. vulgaris*, *K. pneumoniae*, and *P. aeruginosa* (Figure 2). The evaluation showed that cranberry fruit extracts OE, OW, ME, and MW statistically significantly inhibited the in vitro growth of *S. aureus*, *S. epidermidis*, *E. coli*, and *K. pneumoniae* strains compared to the control samples. Meanwhile, the growth of the *P. aeruginosa* strain was statistically significantly reduced by the aqueous MW extract of *V. macrocarpon* fruit. The concentrations of OE, OW, ME, and MW extracts did not affect the growth of *P. vulgaris* strains when compared to the results of the control test (*p* > 0.05).

The bacterial cultures tested can be arranged in the following order according to their susceptibility to cranberry fruit extracts: Gram-positive *S. epidermidis* > Gram-positive *S. aureus* and Gram-negative *E. coli* > Gram-negative *K. pneumoniae* > Gram-negative *P. vulgaris* and *P. aeruginosa*. Nowak et al. found that cranberry fruit extracts inhibited the growth of Gram-positive bacteria more strongly than they did the growth of Gram-negative bacteria [50]. Other authors reported similar results [51,52,53]. The exact antibacterial mechanisms of cranberry extracts are not clear, but as the cell membranes of Gram-positive bacteria do not contain liposaccharides, they might be more permeable to the active compounds of cranberries compared to the membranes of Gram-negative bacteria [51].

The ability of cranberry extracts to inhibit bacterial growth results from the complex additive or synergistic action of various phytochemicals. The exact mechanisms of action are not fully understood. It is believed that one of the most important factors in the antimicrobial effect of cranberry fruits, their juices, or extracts is the acidic pH created by the acids present in cranberry fruits [50,53]. Acids are partially involved in breaking down the lipopolysaccharide structure of the outer membrane of Gram-negative bacteria, which contributes to their permeability [51]. Lacombe et al. found that phenolic compounds and anthocyanins in cranberry extracts retain antibacterial activity at neutral pH, causing local damage to the cell membrane and leakage of the cytoplasm into the environment [54]. Anthocyanins are less antimicrobially active under neutral pH conditions, possibly due to their instability in this environment [54]. Nowak et al. investigated the effect of cranberry fruit juice with an acidic pH and a neutralized pH on Gram-positive and Gram-negative bacterial cultures [50]. The authors reported that cranberry juice at neutral pH inhibited the growth of all Gram-positive bacteria and many Gram-negative bacteria to a lesser extent than the juice with an acidic pH, while no antibacterial effect was observed against Gram-negative bacterial cultures of *K. pneumonia*, *P. aeruginosa*, or *P. fluorescens* [50].

*V. macrocarpon* fruits are most commonly used for the treatment and prevention of urinary tract infections [52,55]. Uropathogenic *E. coli* is a major etiological factor in many urinary tract infections, causing approximately 85% of cases of cystitis [56]. Other Gram-negative bacteria, such as *Klebsiella pneumoniae*, and some Gram-positive cocci, such as *staphylococci* and *enterococci*, have been implicated in the etiopathogenesis of another 25% of cases of cystitis [56]. The antibacterial effect of cranberries is attributed to the inhibition of bacterial adhesion to the walls of the urinary tract. This effect is mainly attributed to the A-type proanthocyanidins found in *V. macrocarpon* fruits, which are generally considered to be the main parameter of bacterial adhesion-preventing activity, but the molecular mechanism is not yet fully understood [24,57].

Proanthocyanidins account for 63–71% of the total phenolic compounds in cranberry fruit [58]. Junger et al. pointed out that the qualitative and quantitative composition of proanthocyanidins in *V. macrocarpon* and *V. oxycoccos* cranberry fruit differed: the content of the A-type trimeric proanthocyanidins was 15 times lower in the fruit of *V. oxycoccos* than in the fruit of *V. macrocarpon*, which could result in different anti-bacterial actions of this fruit [24]. In our study, there were no statistically significant differences between the proanthocyanidin-containing aqueous OW and MW extracts. This might be due to the fact that the antibacterial activity is strongly influenced by other compounds such as anthocyanins, flavonols, and acids. Rodríguez-Pérez et al. investigated the effect of cranberry extract fractions on the surface hydrophobicity and biofilm formation of uropathogenic *E. coli* and found that, in addition to proanthocyanidins, other compounds, mainly flavonoids, were active against the biofilm formation of *E. coli* and also modified the hydrophobicity of the bacterial surface in vitro [59].

The antimicrobial activity of *V. macrocarpon* ethanolic extract ME against the bacterial strains *S. aureus*, *S. epidermidis*, and *E. coli* was found to be statistically significantly weaker than that of *V. macrocarpon* aqueous extract MW. Similar results were reported by other authors, who found that aqueous *V. macrocarpon* pomace extract and 40% ethanolic cranberry extract had a stronger effect on these bacteria than 96% ethanolic cranberry pomace extract [51].

The antimicrobial activity of the ethanolic (OE) and aqueous (OW) extracts of *V. oxycoccos* cranberries against the tested bacterial cultures was not statistically significantly different (*p* > 0.05). Stobnica et al. investigated the antibacterial activity of aqueous, 40% ethanolic, and 96% ethanolic extracts of *V. oxycoccos* fruit and found that the 40% ethanolic extract had a broader spectrum of antibacterial activity than the aqueous or 96% ethanolic extracts [53].

Different cranberry species and their extracts have different qualitative and quantitative compositions of biologically active compounds, but the in vitro antibacterial effects of the extracts of the compounds tested were similar. More detailed studies are needed to better understand the mechanisms of the antibacterial action of the bioactive compounds in cranberry fruit.

### 3.3. In Vitro Anticancer Activity of Cranberry Fruit Extracts

In order to better understand the anticancer potential of biologically active compounds in *V. oxycoccos* and *V. macrocarpon* fruits, it is relevant to carry out comparative studies of the anticancer activity of different cranberry extracts. In this study, we determined the cytotoxic effects of cranberry fruit extracts OE, OW, ME, and MW in human prostate carcinoma (PPC-1) and renal carcinoma (CaKi-1) cell lines.

**Figure 3:**
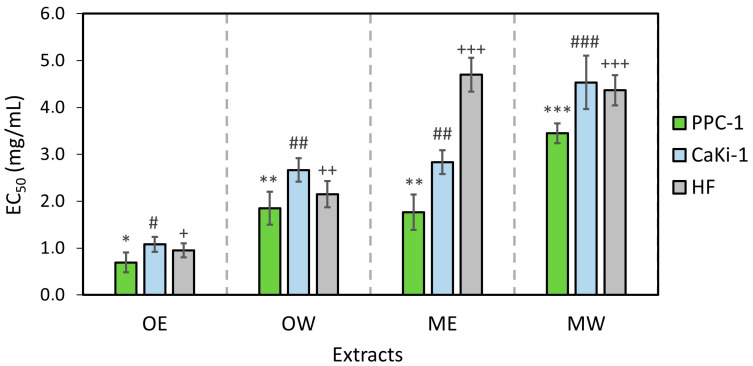
The effect of cranberry extracts on cell line viability using the MTT method. PPC-1—prostate carcinoma cell line; CaKi-1—kidney carcinoma cell line; HF—human skin fibroblast cell line. OE—dry *V. oxycoccos* fruit extract obtained by extraction with 96% (*v*/*v*) ethanol; OW—dry *V. oxycoccos* fruit extract obtained by extraction with hot water; ME—dry *V. macrocarpon* fruit extract obtained by extraction with 96% (*v*/*v*) ethanol; MW—dry *V. macrocarpon* fruit extract obtained by extraction with hot water. Statistically significant differences between the cytotoxic activity of cranberry extracts are indicated by different marks (*/^#^/^+^/**/^##^/^++^/***/^###^/^+++^ *p* < 0.05).

Aqueous and ethanolic extracts of *V. oxycoccos* and *V. macrocarpon* fruits reduced the viability of both prostate carcinoma (PPC-1) and renal carcinoma (CaKi-1) cell lines (Figure 3). The tested cranberry fruit extracts OE, OW, ME, and MW showed 1.3–1.6 times higher cytotoxic activity in PPC-1 cell lines than in CaKi-1 cell lines. The ethanolic V*. oxycoccos* fruit extract OE showed the most pronounced antitumor activity among the cranberry fruit extracts tested (EC_50_ values in the PPC-1 and CaKi-1 cell lines were 0.69 ± 0.21 mg/mL and 1.08 ± 0.16 mg/mL, respectively). Meanwhile, the aqueous *V. macrocarpon* fruit extract MW showed the weakest anticancer activity (EC_50_ values for PPC-1 and CaKi-1 cell lines were 3.45 ± 0.21 mg/mL and 4.53 ± 0.57 mg/mL, respectively).

The study evaluated the effect of aqueous and ethanolic *V. oxycoccos* and *V. macrocarpon* fruit extracts on the viability of non-cancerous human fibroblast (HF) cell lines. The results obtained are important for determining the selectivity of the tested extracts for cancer cells compared to their effect on non-cancerous cells. We found that cranberry fruit sample extracts OE, OW, and MW were non-selective in inhibiting the viability of non-cancerous human fibroblast (HF) cell lines, compared to the viability of cancerous prostate carcinoma (PPC-1) and renal carcinoma (CaKi-1) cell lines (*p* > 0.05). Meanwhile, the ethanolic (ME) extract of *V. macrocarpon* fruit samples had a weaker effect on the viability of human fibroblast (HF) cell lines than on PPC-1 and CaKi-1 cancer cell lines (*p* < 0.05). The interpretation of the results suggests that the ethanolic cranberry fruit extract (ME) had a more selective effect on the viability of cancerous PPC-1 and CaKi-1 cell lines than on the viability of non-cancerous HF cell lines when compared with the effects of the cranberry fruit sample extracts OE, OW, and MW.

**Figure 4:**
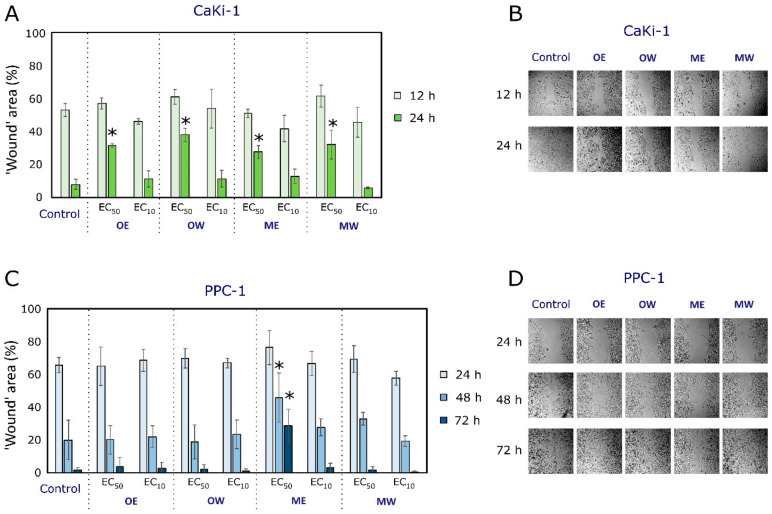
Study of the effect of cranberry extracts on cell migration activity using the ‘wound healing’ method. (**A**) Quantitative calculations of the ‘wound’ area (%) of CaKi-1 cell line monolayer. (**B**) Photos of the ‘wound’ area in the CaKi-1 monolayer after 12 and 24 h of incubation with cranberry extracts and control. (**C**) Quantitative calculations of the ‘wound’ area (%) of PPC-1 cell line monolayer. (**D**) Photos of the ‘wound’ area in the PPC-1 monolayer after 24,48 and 72 h of incubation with cranberry extracts and control. PPC-1—prostate carcinoma cell line; CaKi-1—kidney carcinoma cell line. OE—dry *V. oxycoccos* fruit extract obtained by extraction with 96% (*v*/*v*) ethanol; OW—dry *V. oxycoccos* fruit extract obtained by extraction with hot water; ME—dry *V. macrocarpon* fruit extract obtained by extraction with 96% (*v*/*v*) ethanol; MW—dry *V. macrocarpon* fruit extract obtained by extraction with hot water. Statistically significant differences between the migration activity of cranberry extracts are indicated by marks (* *p* < 0.05).

The effect of cranberry fruit extracts (OE, OW, ME, and MW) on the migration of PPC-1 and CaKi-1 cancer cells was evaluated using the “wound healing” method at EC_10_ and EC_50_ concentrations of the extracts (Figure 4). The EC_10_ concentrations of the cranberry fruit extracts did not have a statistically significant effect on the migration of PPC-1 or CaKi-1 in cancer cells compared to the control. The EC_50_ concentrations of cranberry fruit extracts (OE, OW, ME, and MW) did not have an effect on the CaKi-1 migration activity after 12 h. Still, they statistically significantly decreased it after 24 h, compared with the control samples.

The EC_50_ concentrations of cranberry fruit extracts OE, OW, and MW did not have a statistically significant effect on the migratory activity of PPC-1 cells after 24, 48, or 72 h, compared to the control. Meanwhile, we found that the ethanolic *V. macrocarpon* extract ME at EC_50_ concentration statistically significantly inhibited the migration of the PPC-1 cell line after 48 and 72 h.

**Figure 5:**
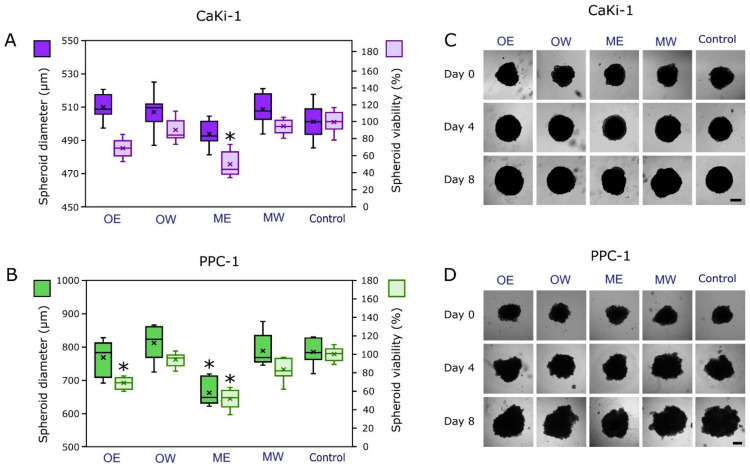
The effect of cranberry extracts on spheroid diameter and viability using the magnetic 3D bioprinting method. (**A**) CaKi-1 spheroid size and viability of cells in spheroids at the end of the experiment. (**B**) PPC-1 spheroid size and viability of cells in spheroids at the end of the experiment. (**C**) Photos of CaKi-1 tumor spheroids at the beginning (Day 0), in the middle (Day 4), and at the end (Day 8) of the experiment. (**D**) Photos of PPC-1 tumor spheroids at the beginning (Day 0), in the middle (Day 4), and at the end (Day 8) of the experiment. PPC-1—prostate carcinoma cell line; CaKi-1—kidney carcinoma cell line. OE—dry *V. oxycoccos* fruit extract obtained by extraction with 96% (*v*/*v*) ethanol; OW—dry *V. oxycoccos* fruit extract obtained by extraction with hot water; ME—dry *V. macrocarpon* fruit extract obtained by extraction with 96% (*v*/*v*) ethanol; MW—dry *V. macrocarpon* fruit extract obtained by extraction with hot water. Statistically significant differences between the spheroid diameter and viability of cranberry extracts are indicated by marks (* *p* < 0.05).

During the study, the effect of cranberry extracts on the CaKi-1 and PPC-1 spheroid growth and viability were evaluated using the magnetic 3D bioprinting method (Figure 5). A 3D cell model can better mimic the tumor microenvironment than conventional cell monolayers [60]. Only ethanolic *V. macrocarpon* (ME) extract significantly inhibited the viability of CaKi-1 spheroids by 45.0% compared to the control. The viability of PPC-1 spheroids was significantly inhibited by ethanolic extracts of both *V. macrocarpon* (ME) and *V. oxycoccos* (OE) (by 49.1% and 31.9%, respectively, compared to the control). Cranberry extract did not affect CaKi-1 spheroid growth, while PPC-1 spheroid growth was statistically significantly delayed only by ethanolic *V. macrocarpon* (ME).

Literature data have shown that *V. macrocarpon* fruit extracts inhibited the viability of breast, colon, prostate, lung, kidney, stomach, and melanoma tumor cell lines in vitro [61]. Given the fact that cranberry fruit has been found to contain different bioactive compounds, different potential anticancer mechanisms can be identified. The compounds identified in cranberry fruit (mainly proanthocyanidins, triterpene compounds, and quercetin) inhibit tumor cell viability, promote apoptosis, inhibit proliferation and colony formation, and limit the ability of cells to migrate and metastasize [61,62]. Bioactive compounds of cranberry fruit have been shown to reduce ornithine decarboxylase activity and the expression of matrix metalloproteinases, and they also exert anti-inflammatory effects by inhibiting the activity of cyclooxygenases [62].

The cytotoxic effects of our studied cranberry fruit extracts (OE, OW, ME, and MW) on prostate carcinoma (PPC-1) extracts of cranberry fruit were different, indicating that the anticancer effect was due to different groups of bioactive compounds. Similar results were obtained by other investigators, showing that different groups of cranberry fruit compounds, such as proanthocyanidins, flavonols, and ursolic acid and its derivatives, exhibit anticancer activity [16,63].

The anticancer activity of the ethanolic cranberry fruit extracts OE and ME was attributed to the triterpene compounds detected therein. Ursolic and oleanolic acids were the predominant triterpenoids in cranberry fruit samples and accounted for 74–76% and 20–22% of the triterpenoid compounds detected in the ethanolic extracts tested, respectively. The anticancer activity of ursolic and oleanolic acids has been established against various cell lines [64]. Cai et al. found that ursolic acid inhibited the migratory activity of CaKi-1 cells, reduced the ability of CaKi-1 cells to form renal tubules, and reduced the volume and weight of CaKi-1 cell tumors [65]. Vilkickytė et al. investigated the fractions of triterpene compounds isolated from lingonberry fruit (*Vaccinium vitis-idea* L.) and found that the fraction consisting of oleanolic and ursolic acids exhibited the strongest cytotoxic activity against the CaKi-1 cell line (the EC_50_ was 4.5 µg/mL) [66].

Kondo et al. reported that ursolic acid and its cis- and trans-3-O-p-hydroxycinnamoyl esters found in *V. macrocarpon* fruits inhibited the proliferation of breast, prostate, lung, colorectal, leukemia, and kidney tumor cells [16]. The study conducted by these researchers revealed the effect of the isolated compounds (ursolic acid and cis- and trans-3-O-p-hydroxycinnamoyl esters of ursolic acid) on cell viability and matrix metalloproteinase (MMP) expression in the DU145 prostate tumor cell line [16]. All compounds were found to inhibit the activity of MMP-2 and MMP-9 matrix metalloproteinases, and cis- and trans-3-O-p-hydroxycinnamoyl ursolic acid esters were found to be more potent than ursolic acid at 10 μg/mL [16]. The authors reported that the content of cis- and trans-3-O-p-hydroxycinnamoyl ursolic acid esters in *V. oxycoccos* fruits was significantly lower (by 13–17 and 210–300 times, respectively) than in *V. macrocarpon* fruits [16]. We did not detect these specific ursolic acid esters in extracts OE or ME, but it is likely that these compounds may play an important role in the activity of the metalloproteinases MMP-2 and MMP-9, which are involved in the invasion and metastasis of prostate tumor cells. This could explain why the ethanolic *V. macrocarpon* extract ME showed a migration-inhibiting effect and spheroid growth inhibition in the PPC-1 cell line, whereas this effect was not observed with the ethanolic extract of *V. oxycoccos* (OE). Further studies on the phytochemical composition and anticancer activity are needed to confirm this hypothesis.

The anticancer activity of the aqueous cranberry fruit extracts OW and MW might be attributed to their flavonoid content. Seeram et al. found that a water-soluble polyphenol extract of *V. macrocarpon* inhibited the viability of oral (CAL27 and KB), colon (HT-29, HCT-116, SW480, and SW620), and prostate (RWPE-1, RWPE-2, and 22Rv1) cancer cell lines more potently than the individual fractions of anthocyanins, proanthocyanidins, or flavonols [63]. This suggests that additive or synergistic interactions between anthocyanins, proanthocyanidins, and flavonol glycosides in their antiproliferative effects are important in the mechanisms of the anticancer activity of bioactive compounds in cranberry fruit [67].

PACs and other flavonoids found in cranberry fruits are capable of inhibiting cell viability and processes related to tumor invasion and metastasis by suppressing the expression of matrix metalloproteinases [62,68]. Neto et al. found that proanthocyanidin fractions in cranberry fruit inhibited the viability of the prostate cancer cell lines PC3 and DU-145 (GI50 125 µg/mL), but not in a selective manner, compared to a non-neoplastic fibroblast cell line in mice (GI50 125 µg/mL) [69]. The cranberry PAC fraction also inhibited MMP expression in the DU-145 cell line, but to a lesser extent than the whole *V. macrocarpon* fruit extract [69]. MacLean et al. found that DU145 cell apoptosis in vitro induced by bioactive compounds in cranberry fruit may be due to increased caspase-8 and caspase-9 activity [70].

Experimental in vivo and in vitro studies have been conducted to determine the anticancer activity of quercetin and its glycosides [71]. Flavonols can act on cancer cells by inhibiting cell proliferation, promoting apoptosis, inhibiting angiogenesis and the progression of metastases, and affecting autophagy [72]. Vilkickyte et al. investigated the cytotoxic effects of flavonols and proanthocyanidins on cancer cells and found that quercetin and procyanidin-A2 exhibited the most potent anticancer activity against the renal (CaKi-1) cancer cell line (EC_50_ values of 40.8 µM and 63.0 µM, respectively) [73].

Neto et al. found that a cranberry polyphenolic extract at a concentration of 100 µg/mL inhibited the expression of matrix metalloproteinases MMP-2 and MMP-9 in the DU-145 prostate tumor cell line by 20% and 10%, respectively [69]. Ursolic acid at a concentration of 10 µg/mL inhibited the expression of matrix metalloproteinases MMP-2 and MMP-9 in DU-145 prostate tumor cell lines by 80% and 20%, respectively [16]. This suggests that triterpene compounds have a stronger inhibitory effect on the expression of matrix metalloproteinases.

The studied cranberry extracts (OE, OW, ME, and MW) contained anthocyanins, which may be involved in cancer-inhibiting mechanisms. Seeran et al. found that the subfraction of cranberry anthocyanin limited the growth of prostate tumor cell lines (RWPE-1, RWPE-2, and 22Rv1) by 55–69% but did not significantly inhibit the proliferation of oral or colorectal tumor cell lines [63]. Anthocyanin-containing extracts inhibit the induction of vascular endothelial growth factor (VEGF), suggesting that the antioxidant and anti-inflammatory properties of these compounds may limit angiogenesis [62]. The antioxidant properties of cranberry anthocyanins may inhibit oxidative processes associated with tumorigenesis, but the direct antiproliferative or growth-inhibiting effects of anthocyanins in vitro models have been found to be generally low in the cell lines studied [14].

*V. oxycoccos* and *V. macrocarpon* fruit extracts showed inhibitory effects on the viability of prostate and kidney cancer cell lines. The ethanolic *V. macrocarpon* fruit extract (ME) showed a more selective inhibitory effect on the viability of prostate and renal cancer cell lines compared to fibroblasts and also inhibited the migration of these cancer cell lines. In addition, the *V. macrocarpon* fruit extract (ME) showed the strongest cytotoxic activity on PPC-1 and CaKi-1 spheroids and statistically significantly reduced the size of PPC-1 spheroids compared to the control. Given the fact that extracts with different phytochemical compositions reduce cell viability, it is relevant to further investigate the individual effects and synergistic interactions of bioactive compounds.

## 4. Conclusions

This study aimed to determine the phytochemical composition of extracts from *V. oxycoccos* and *V. macrocarpon* fruits and assess their antioxidant, antibacterial, and anticancer properties in vitro. The study revealed that the ethanolic extracts of *V. oxycoccos* and *V. macrocarpon* fruits exhibited lower antioxidant activity than the dry aqueous extracts of cranberry fruit. The antibacterial effects of the tested cranberry extracts were similar, despite the identified differences in the phytochemical composition of the extracts. The ethanolic extract from *V. macrocarpon* fruits exhibited higher selectivity in inhibiting prostate and renal cancer cell viability while also impeding their migration. Moreover, ethanolic extract from *V. macrocarpon* fruits demonstrated potent cytotoxicity against PPC-1 and CaKi-1 spheroids compared to the control. Based on these findings, future research could focus on elucidating the specific compounds responsible for the observed anticancer and antibacterial effects, as well as investigating the potential interactions of cranberry fruit extracts with conventional therapies, thereby contributing to the development of new treatments for bacterial infections and cancer.

## Figures and Tables

**Table 1:** Phytochemical composition of dried cranberry fruit extracts.

Compound	OE	OW	ME	MW
Proanthocyanidins	-	1531 ± 46 ^a^	-	1359 ± 119 ^b^
Chlorogenic acid	86.4 ± 0.5 ^c^	1897.3 ± 18.9 ^a^	6.6 ± 0.1 ^d^	458.2 ± 4.6 ^b^
Flavonols				
Myricetin-3-galactoside	-	753.7 ± 4.3 ^a^	-	680.0 ± 3.9 ^b^
Quercetin-3-galactoside	8.8 ± 0.1 ^d^	850.2 ± 8.5 ^b^	54.2 ± 0.5 ^c^	1236.2 ± 12.3 ^a^
Quercetin-3-glucoside	-	126.3 ± 1.3 ^a^	-	34.1 ± 0.4 ^b^
Quercetin-3-α-L-arabinopyranoside	-	94.1 ± 1.0 ^a^	-	99.1 ± 1.0 ^a^
Quercetin-3-α-L-arabinofuranoside	4.8 ± 0.1 ^d^	195.2 ± 2.0 ^b^	36.5 ± 0.4 ^c^	532.2 ± 5.6 ^a^
Quercetin-3-rhamnoside	19.2 ± 0.2 ^d^	121.1 ± 1.3 ^b^	51.2 ± 0.5 ^c^	250. ± 2.7 ^a^
Quercetin	4.0 ± 0.0 ^d^	78.7 ± 0.6 ^a^	13.6 ± 0.1 ^c^	17.2 ± 0.1 ^b^
Myricetin	8.9 ± 0.1 ^d^	67.9 ± 0.5 ^a^	13.6 ± 0.1 ^c^	15.0 ± 0.1 ^b^
Sum of flavonols	45.7 ± 0.4 ^d^	2287.3 ± 19.5 ^b^	169.1 ± 1.7 ^c^	2864.6 ± 26.1 ^a^
Anthocyanins and anthocyanidins				
Delphinidin-3-galactoside	6.9 ± 0.1 ^d^	18.0 ± 0.2 ^a^	8.2 ± 0.1 ^c^	12.7 ± 0.1 ^b^
Cyanidin-3-galactoside	131.3 ± 2.6 ^d^	1092.9 ± 21.9 ^b^	149.8 ± 3.0 ^c^	1734.0 ± 34.7 ^a^
Cyanidin-3-glucoside	42.9 ± 1.0 ^b^	174.7 ± 4.0 ^a^	11.9 ± 0.3 ^d^	38.5 ± 0.9 ^c^
Cyanidin-3-arabinoside	19.5 ± 0.3 ^d^	952.8 ± 12.5 ^b^	29.8 ± 0.4 ^c^	1116.7 ± 14.6 ^a^
Peonidin-3-galactoside	197.5 ± 2.1 ^d^	1370.1 ± 14.4 ^b^	269.0 ± 2.8 ^c^	2329.6 ± 24.6 ^a^
Peonidin-3-glucoside	153.6 ± 1.8 ^b^	395.1 ± 4.8 ^a^	52.7 ± 0.6 ^d^	124.2 ± 1.5 ^c^
Peonidin-3-arabinoside	8.3 ± 0.1 ^c^	688.3 ± 8.3 ^b^	-	786.8 ± 8.8 ^a^
Malvidin-3-galactoside	21.3 ± 0.3 ^a^	11.7 ± 0.1 ^b^	3.3 ± 0.0 ^c^	11.5 ± 0.1 ^b^
Malvidin-3-arabinoside	-	16.9 ± 0.2 ^a^	-	9.1 ± 0.1 ^b^
Cyanidin chloride	495.9 ± 5.5 ^b^	28.5 ± 0.3 ^c^	1361.6 ± 13.6 ^a^	9.9 ± 0.1 ^d^
Peonidin chloride	902.3 ± 10.4 ^b^	23.8 ± 0.3 ^c^	2121.1 ± 24.4 ^a^	6.1 ± 0.1 ^d^
Malvidin chloride	249.9 ± 3.6 ^b^	-	304.3 ± 4.4 ^a^	-
Sum of anthocyanins and anthocyanidins	2229.4 ± 27.7 ^d^	4773.0 ± 66.8 ^b^	4311.8 ± 49.7 ^c^	6179.1 ± 85.6 ^a^
Triterpenoids				
Maslinic acid	425.1 ± 5.6 ^a^	-	13.1 ± 0.2 ^b^	-
Corosolic acid	57.2 ± 1.0 ^b^	-	109.6 ± 1.9 ^a^	-
Oleanolic acid	1422.7 ± 18.6 ^a^	-	913.9 ± 12.0 ^b^	-
Ursolic acid	5220.8 ± 81.0 ^a^	-	3047.1 ± 47.3 ^b^	-
Sum of triterpenoids	7125.9 ± 106.2 ^a^	-	4083.6 ± 61.3 ^b^	-
β-sitosterol	742.9 ± 10.9 ^b^	386.7 ± 5.7 ^d^	793.1 ± 11.7 ^a^	560.0 ± 8.2 ^c^

The amount of compounds is expressed in µg/g. OE—dry *V. oxycoccos* fruit extract obtained by extraction with 96% (*v*/*v*) ethanol; OW—dry *V. oxycoccos* fruit extract obtained by extraction with hot water; ME—dry *V. macrocarpon* fruit extract obtained by extraction with 96% (*v*/*v*) ethanol; MW—dry *V. macrocarpon* fruit extract obtained by extraction with hot water. Statistically significant differences between the amounts of compounds determined in cranberry extracts are indicated by different letters (*p* < 0.05).

## Data Availability

Data are contained within the article.
